# Distribution of Mosquitoes in the South East of Argentina and First Report on the Analysis Based on 18S rDNA and COI Sequences

**DOI:** 10.1371/journal.pone.0075516

**Published:** 2013-09-30

**Authors:** Leonardo M. Díaz-Nieto, Arnaldo Maciá, Gustavo Parisi, Juan L. Farina, María E. Vidal-Domínguez, M. Alejandra Perotti, Corina M. Berón

**Affiliations:** 1 Instituto de Investigaciones en Biodiversidad y Biotecnología (INBIOTEC), CONICET, Mar del Plata, Argentina; 2 División Entomología, Facultad de Ciencias Naturales y Museo Universidad Nacional de La Plata, La Plata, Argentina; 3 Departamento de Ciência y Tecnología, Universidad Nacional de Quilmes, Buenos Aires, Argentina; 4 Area Entomología, Museo Municipal de Ciencias Naturales "Lorenzo Scaglia", Mar del Plata, Argentina; 5 Ecology and Evolutionary Biology Section, School of Biological Sciences, University of Reading, Reading, Berkshire, United Kingdom; Virginia Tech, United States of America

## Abstract

Although Mar del Plata is the most important city on the Atlantic coast of Argentina, mosquitoes inhabiting such area are almost uncharacterized. To increase our knowledge in their distribution, we sampled specimens of natural populations. After the morphological identification based on taxonomic keys, sequences of DNA from small ribosomal subunit (18S rDNA) and cytochrome *c* oxidase I (COI) genes were obtained from native species and the phylogenetic analysis of these sequences were done. Fourteen species from the genera *Uranotaenia*, *Culex* , *Ochlerotatus* and *Psorophora* were found and identified. Our 18S rDNA and COI-based analysis indicates the relationships among groups at the supra-species level in concordance with mosquito taxonomy. The introduction and spread of vectors and diseases carried by them are not known in Mar del Plata, but some of the species found in this study were reported as pathogen vectors.

## Introduction

Humid tropics and subtropics are the natural geographical range for approximately three quarters of all mosquito species, although these insects may also cause a considerable problem in temperate latitudes. This area of the world is especially prone to suffer the consequences of global warming [1], including a rise in health risks due to an expansion of the distribution of vectors of diseases like dengue, yellow fever, malaria, lymphatic filariasis and several types of encephalitis like West Nile fever [2]. To carry on the correct identification of mosquito vectors and their geographic distribution is a critical step, as well as the active monitoring and surveillance of mosquito populations. Although the identification of mosquito species based on their external anatomy is sometimes difficult, time-consuming, and often limited to adult females or fourth instar larvae, is remains as the most accepted methodology. Morphological characteristics are often insufficient to differentiate between species, and ecological, genetic, distributional or behavioral features must be taken into account when sibling species are to be detected. To overcome the difficulties associated with species identification, genetic characters using molecular methodologies could provide reliable means of taxonomic discrimination and have emerged as very helpful and complementary tools in identifying insects. Several biochemical and molecular approaches have been applied in order to identify mosquito species [3,4]. Among them, identification systems based on DNA nucleotide sequence analysis is one of the most well-known and widely applied methodologies. The small subunit 18S rDNA gene is one of the most frequently used genes in phylogenetic studies and is an important marker for environmental biodiversity screening. Gene sequences obtained from 18S rDNA have been successfully used to examine the evolutionary relationships among species, genera, and higher taxonomic groups of different insects [4]. However, the use of 18S rDNA sequence analysis for evaluating phylogenetic relationships among some mosquito species has shown some limitations [5]. On the other hand, the analysis of sequence variation in the 5′ region of the mitochondrial cytochrome *c* oxidase I (COI) gene (DNA barcoding) promises fast, accurate species identifications by focusing analysis on a short standardized segment of the genome. Several studies have now established that sequence diversity in a 650-bp fragment of the COI gene provides strong species-level resolution [6] and also, its effectiveness in discrimination of mosquito species has been recognized [7,8]. Using molecular techniques as a complementary tool alongside current morphological identification systems have the potential to improve the speed and accuracy of mosquito identification practices.

There are important gaps in the knowledge about identity and distribution of mosquitoes in certain areas of Argentina. For example, in the subgenus *Culex* (Culex), in spite of being the most diverse of the genera, the identification of the species according to certain publications is based on features that are sometimes subjective and not very reliable. This lack of appropriate knowledge about the mosquito fauna is evident in some localities such as General Pueyrredon district in Buenos Aires province, Argentina, being Mar del Plata its main city. Mar del Plata is the most important beach resort in Argentina having a stable population of about 650,000 inhabitants, and nearly 8 million tourists visit the city throughout the year. At least 2 million people arrive during the summer season, principally from Gran Buenos Aires, the most populated area in the country with 12,801,365 residents, representing 32% of the total population of Argentina (www.censo2010.indec.gov.ar; http://www.turismomardelplata.gov.ar). Despite the importance of this large urban center not much is known about culicids from this part of the country. Our purpose was to fill this lack of information about local species through sampling natural populations and then to identify those using taxonomic keys and molecular techniques.

Herein, we evaluated the identity of mosquitoes from General Pueyrredon district, Buenos Aires, Argentina based on classical taxonomy for the first time. On the other hand, we explored the usefulness and constraints of 18S rDNA and COI sequences for taxonomic purposes in local mosquito species, as well as the correlation with their bionomics. We present the first sequences of 18S rDNA and COI genes of species from Argentina in order to make them available for comparative studies.

## Materials and Methods

### Ethics Statement

This work was carried out under scientific license provided by the OPDS (Provincial Agency for Sustainable Development) for sampling in protected areas, in some private places where specific permits from the owners were needed, and in some free-access lands where permits were not required. None of the endangered or protected species were included in this study.

### Sampling area

**Figure 1:**
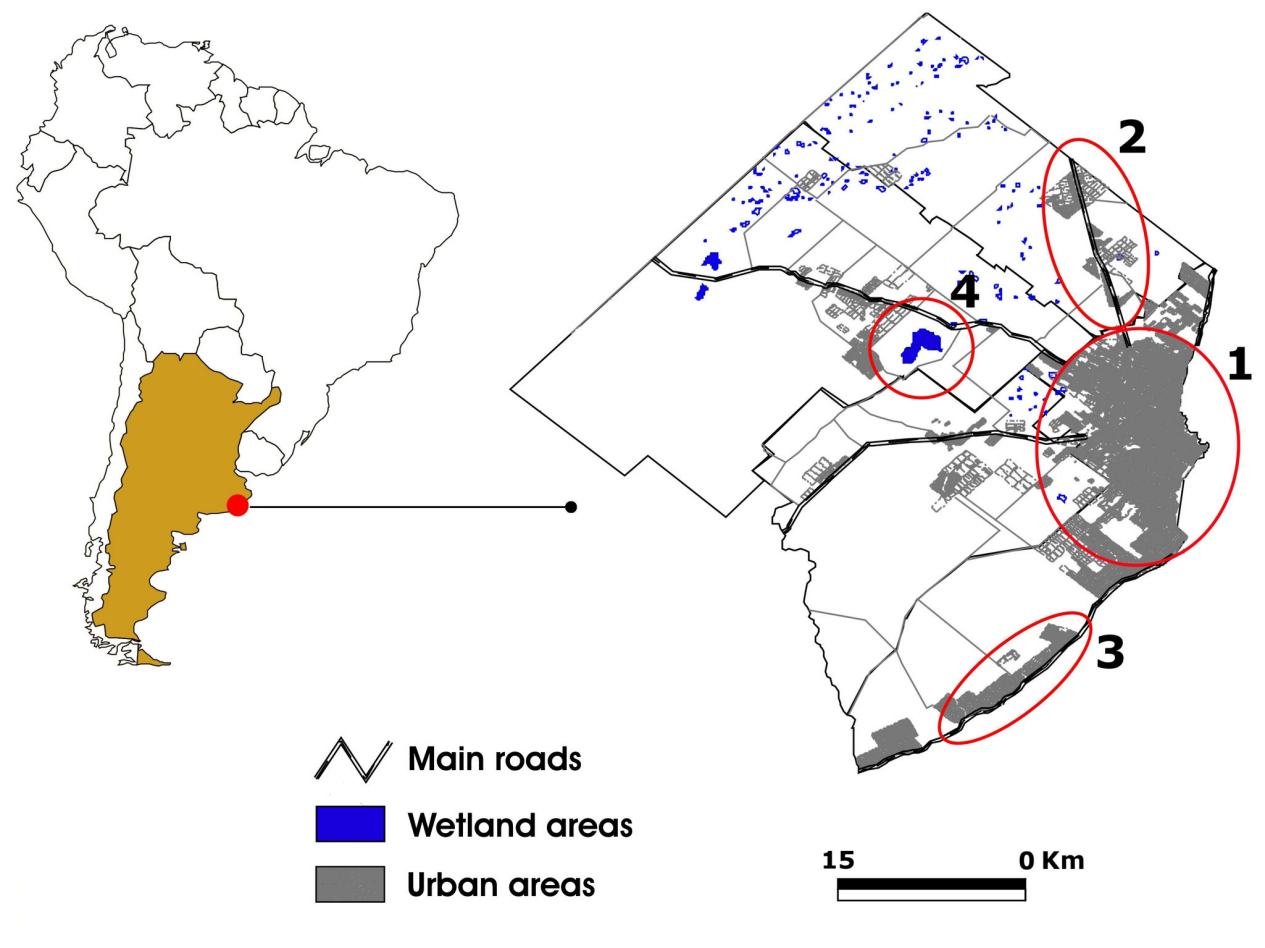
Sampling area and freshwater habitats from General Pueyrredon municipality. 1, Urban area with natural and artificial containers; 2, roads towards Mar del Plata with puddles alongside; 3 roads in coastal area with puddles alongside; 4, area of nature reserve with wetlands, freshwater swamps and bogs.

Mosquitoes were collected in General Pueyrredon district (Figure 1), located on the coast of the Atlantic Ocean, in the South-East of Buenos Aires province (38° 00´ S, 57°33´ W). Its total surface accounts for 1,453.44 km^2^ of which 79.48 km^2^ are taken up by the Mar del Plata urban area. This geographic region is characterized by a great diversity of biotopes, most of which have favorable conditions for the development of mosquitoes. The area is crossed from west to east by numerous streams which rise in the highlands of the province. There are numerous wetlands like “Reserva Integral Laguna de los Padres” (a provincial reserve) and “Laguna Mar Chiquita” (a Ramsar site) which are permanently visited. The climate is mild with maritime influence and the average temperature is 8°C (46°F) in winter and 20°C (68°F) in summer. Rainfalls are abundant throughout the year, and the weather, according to Köppen climate classification, is similar to Western Europe [9].

### Insect Collections and Identification

**Figure 2:**
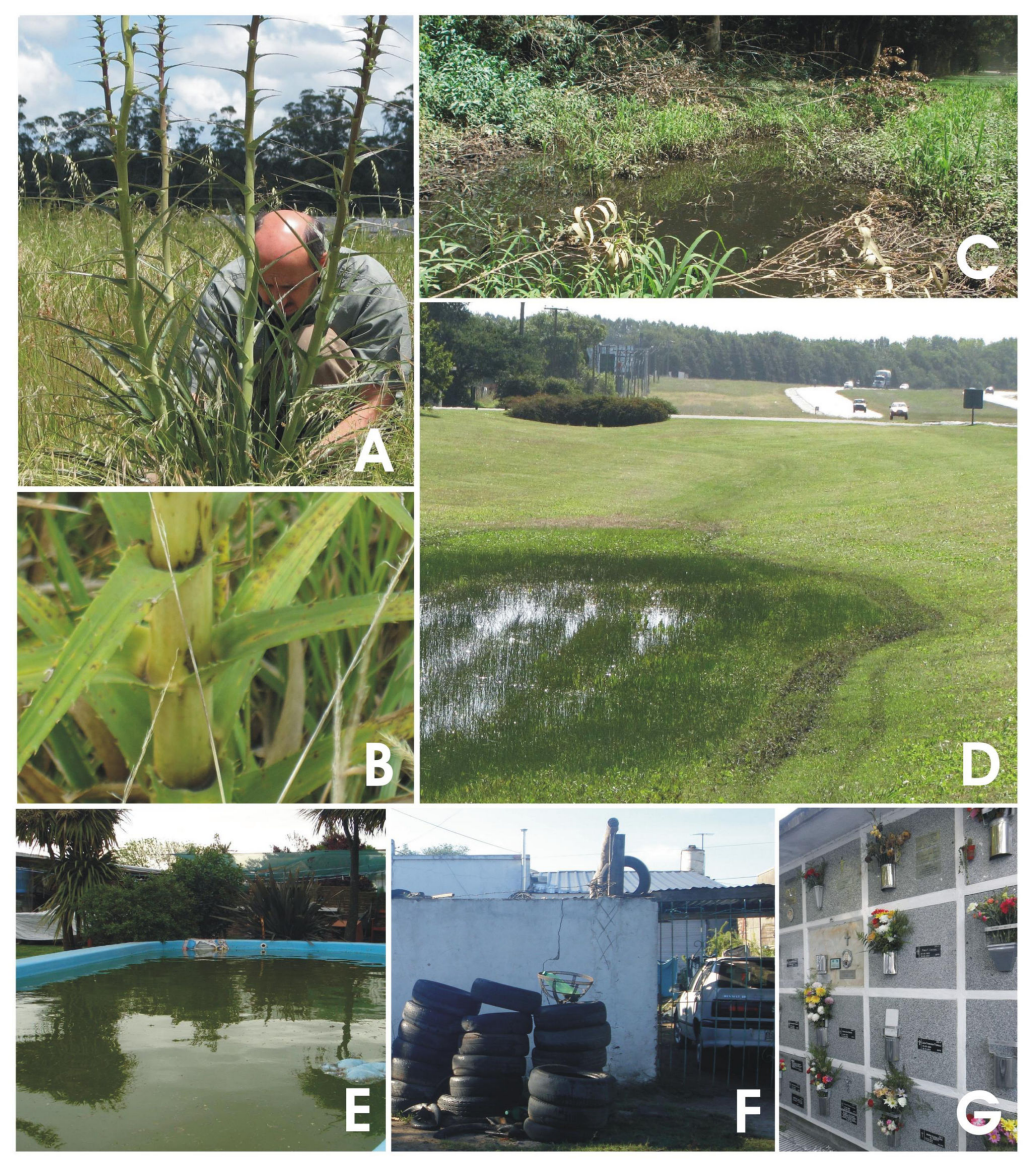
Surveyed mosquito habitats. A, *Eryngium* *horridum* ; B, detail of leaf axils of *E* *. horridum* ; C, freshwater pool in nature reserve; D, puddles along roads; E-G, artificial containers. Dr. Maciá has given written informed consent, as outlined in the PLOS consent form, to publication of this photograph.

Mosquitoes were collected between September 2009 and April 2011. Adult collections were made with a CDC Mini Light Trap with incandescent light (model 2836BQ, BioQuip Products, Rancho Dominguez, California, USA). Hand-held aspirators were used to collect host-seeking females of species that could not be caught in light traps. Larval specimens were collected from vernal pools, freshwater swamps, bogs, and natural and artificial containers (Figure 2), and reared until fourth instar or adulthood. Voucher specimens, prepared from all localities, were submitted to the local museum, Museo de Ciencias Naturales “Lorenzo Scaglia” (Mar del Plata, Argentina). Mosquito fourth instar larvae and adult females were recognized using identification keys by Darsie and Mitchell [10] and Rossi et al. [11]. Specimens were stored at -20°C until DNA extraction.

### Nucleic acid isolation, primer design and PCR conditions

Genomic DNA was isolated from a single mosquito at the fourth larval instar or at the adult stage. After all individuals were sorted by species using taxonomical keys, sampling place and breeding habitat type, one individual of each of these groups was preserved as morphological voucher and another one as a source of genomic DNA. Total DNA was extracted with the PureLink Genomic DNA Mini Kit (Invitrogen, Grand Island, New York, USA) according to the manufacturer’s instructions.

For the amplification of a fragment of the COI the standard primers were useful, and for nuclear 18S rDNA some samples were amplified at a first step by EukA/EukB primers [12], but a new oligonucleotides design from some Culicidae sequences was needed for most of the samples. For that, we designed a set of primers using ClustalW2 Server [13] to compare 18S rDNA sequences of the different Culicidae genera retrieved from Genbank/EMBL databases. Primer sequences and PCR annealing temperature of each are shown in Table 1.

**Table 1:** Primers used for PCR reactions from DNA mosquitoes samples.

**Primer pair**	**Primer sequence ^a^**	**Position**	**Product size (bp)**	**Annealing temperature in PCR reaction**
Cx 2 *f* Cx 1722 *r*	5’ GCCATGCATGTCTAAGTACAAACAGWTTT 3’ 5’ CTTTGTACACACCGCCCGTCGCTAC 3’	2-1722^b^	1745 bp	60°C
Cx 2 *f* Cx 605 *r*	5’ GCCATGCATGTCTAAGTACAAACAGWTTT 3’ 5’ GCTGGAATTACCGCGGCTGCTGG 3'	2-605^b^	544 bp	60°C
Cx 583 *f* Cx 1722 *r*	5' CCAGCAGCCGCGGTAATTCCAGC 3' 5’ CTTTGTACACACCGCCCGTCGCTAC 3’	583-1722^b^	1223 bp	60°C
EukA EukB	5’ AACCTGGTTGATCCTGCCAGT 3’ 5’ TGATCCTTCTGCAGGTTCACCTAC 3’	1-1773^c^	1796 bp	50°C
LCO 1490 HCO 2198	5’ GGTCAACAAATCATAAAGATATTGG 3’ 5’ TAAACTTCAGGGTGACCAAAAAATCA 3’	1490-2198^d^	710 bp	46°C

*f* forward primer, *r* reverse primer

^a^ One of the sequence corresponds to degenerate primers, and are indicated according to the degenerate DNA genetic code as follows: W = T or A

^b^ Position starting from the second base of the available sequence of the 18S rDNA from *C* *. pipiens* *quinquefasciatus* (accession number AY988447) according to sequences obtained from the National Center for Biotechnology Information database

^c^ From the available sequence of the 18S rDNA of *Tetraselmis* *sp* (accession number U41900) according to sequences obtained from the National Center for Biotechnology Information database [12]

^d^ From the available sequence of the 18S rDNA of *Drosophila* *yacuba* (accession number X03240) according to sequences obtained from the National Center for Biotechnology Information database [70]

The PCR reactions were carried out by using standard protocols. The amplified products were analyzed by electrophoresis in 1% (w/v) agarose gels in tris-acetate buffer and ethidium bromide staining [14], and the fragments of about 1,200; 1,800 and 600 bp of 18S rDNA and COI sequences of about 600 bp were purified using Purelink PCR purification kit (Invitrogen) according to the manufacturer’s protocol.

### Sequencing and database analysis of the PCR fragments

The purified PCR product was submitted for direct nucleotide sequencing (Macrogen, Korea). DNA sequence data sets were analyzed by BLASTn and multiple-sequence alignment (Table 2) [15]. Additional 18S rDNA and COI coding-sequences for mosquito species and outgroup taxa were obtained from the National Center for Biotechnology Information (NCBI), http://www.ncbi.nlm.nih.gov (Tables S1 and S2). Retrieved sequences were aligned using ClustalX [16] and phylogenetic analyses were performed. Firstly, DNA alignments were used to estimate the best evolutionary model using the program ModelTest [17]. These models were selected using AIC score [18] and used to obtain Maximum Likelihood trees through the PhyML program [19]. Also, Maximum Parsimony and Distance trees (using Neighbor-Joining method) were obtained using the programs MEGA [20] and HYPHY [21] respectively. Maximum Likelihood and distance trees were obtained using Tamura Nei model [22] with observed frequencies and gamma distribution for rate heterogeneity estimation. Tree support was obtained using a non-parametric bootstrapping (using 500 replicons for Maximum Likelihood estimations and 5,000 for Parsimony and Distance methods) followed by a majority rule consensus to obtain the final trees. Trees obtained from the different methods were compared using TOPD program [23]. Finally, trees were rooted using mid-point protocol with the corresponding outgroups and displayed by the Archaeopteryx program [24].

**Table 2:** Origin, abundance and NCBI accession numbers for 18S rDNA and COI sequences of mosquito species included in this study.

**Number of sample**	**Mosquito species**	**Abundance**	**Stage**	**Reservoir Type**	**Accession number**	
					**18S rDNA**	**COI**	
M57	*Culex* *pipiens* Linnaeus	**+++**	larvae	AC- flower vase, swimming pool, ditches, tires, footprints Intermittent ephemeral puddles	HE600026	HE600030	
M1	* Culex pipiens *		larvae	Laboratory breeding		−	
M3	*Culex* *apicinus* Philippi	**+++**	larvae	AC- Swimming pool, flower vase, tires	−	HE600031	
M2	* Culex apicinus *		larvae	Laboratory breeding	HE600022	−	
M31	*Culex* *brethesi* Dyar	**++**	larvae	Intermittent ephemeral puddles, flowing streams	HE600017	HE605121	
M68	*Culex* *renatoi* Lane & Ramalho	**++**	larvae	NC- leaf axils of *Eryngium*	HE600020	HE605119	
M28	*Culex* *chidesteri* Dyar	**++**	larvae	AC- Ditches Intermittent ephemeral puddles	HE600016	HE600028	
M59A/M45*	*Culex* *eduardoi* Casal & García	**++**	larvae	AC- Ditches Intermittent ephemeral puddles	HE600021	HE605120	
M69A	*Culex* *maxi* Dyar	**+**	larvae	Footprints	HE600018	HE599225	
M97**	*Culex* *dolosus* (Lynch Arribálzaga)	**-**	larvae	Intermittent ephemeral puddles	−	HE600027	
M90	*Ochlerotatusalbisfasciatus* (Macquart)	**+++**	larvae	AC- Ditches Intermittent ephemeral puddles	HE600024	HE599223	
M105	*Ochlerotatuscrinifer* (Theobald)	**++**	larvae	AC- Ditches Intermittent ephemeral puddles	HE600023	HE605113	
10	*Ochlerotatus* *scapularis* (Rondani)	**+**	adult	−	−	−	
11	*Ochlerotatusserratus* (Theobal)	**+**	larvae	AC- Ditches	−	−	
M46/M54*	*Psorophora* *cyanescens* (Coquillett)	**++**	larvae	Intermittent ephemeral puddles	HE600019	HE599224	
13	*Psoropora* *ciliata* (Fabricius)	**+**	adult	−	−	−	
M87B	*Uranotaenia* *lowii* Theobald	**++**	larvae	Marshy areas	HE600025	HE600029	

AC: artificial containers.NC: natural containers.

* Numbers of samples corresponding to 18S rDNA/COI (see trees)

^**^ Sample of La Plata (not belong to Mar del Plata city)

^+++^ > 100 individuals per sample

++: 100 -50 individuals per sample

+: < 50 individuals per sample

The novel sequences reported in this paper have been deposited in the EMBL database (Table 2).

## Results and Discussion

According to the taxonomic keys, we identified 14 mosquito species from four genera. Larvae were recovered from three broad categories of aquatic habitats: freshwater pools, water in axils of plant leaves, and artificial containers. Details of these habitats for each mosquito species are shown in Table 2 and examples in Figure 1. All species found during the sampling period and their abundances are presented in Table 2.

### 18S rDNA Analysis

**Figure 3:**
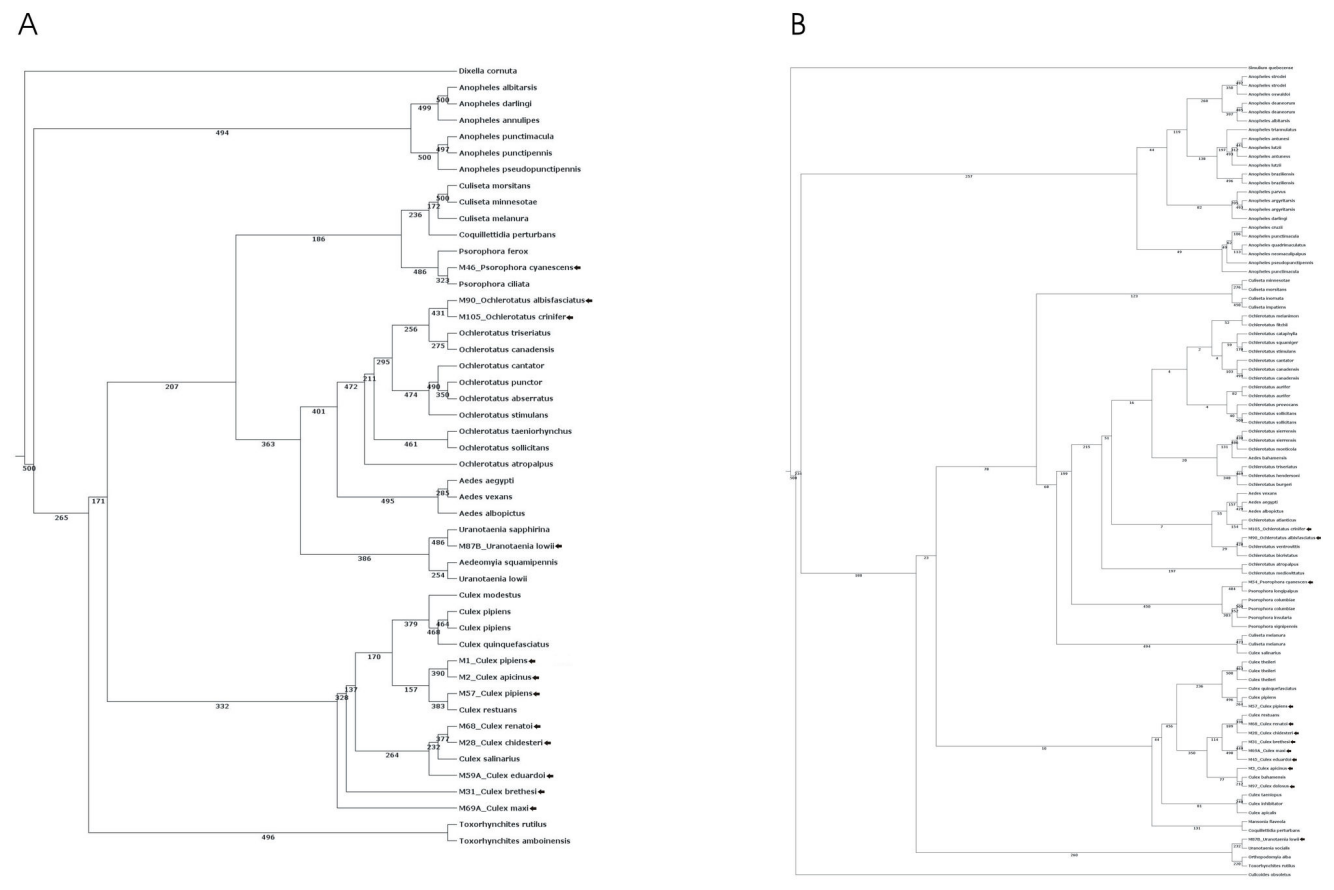
Phylogenetic estimation by Maximum Likelihood. A, Using 18S rDNA; B, COI coding-sequence. 500 replicates have been used in the estimation. Arrows indicates local mosquito species.

Maximum Likelihood, Parsimony and Neighbor-Joining trees based on the information given by mosquito 18S rDNA sequences resulted in very similar trees, with slight differences in the position of species within each genus (Figure 3A and S1). This global topological similarity was also confirmed using the algorithm TOPD showing the robustness of the evolutionary estimation made with different methods. Trees sustained subfamilies (Anophelinae and Culicinae) traditionally accepted for the family Culicidae. *Anopheles* species produced a basal group to the rest of the family members. *Toxorhynchites* stemmed in a basal clade with Culicinae, upholding Toxorhynchitini as a separate tribe but not as a subfamily, as proposed by Harbach and Kitching [25]. As expected, *Dixella* *cornuta* resulted in an external position with respect to Culicidae.

Monophyly was supported for the genus *Culex* with well bootstrapping value and in all the three phylogenies obtained for 18S rDNA (Figure 3A and S1). Members of the other tribes were not classified according to this hierarchical level. Our trees do not support the status of *Ochlerotatus* as separate genera from *Aedes*, as stated by Reinert [26], as seen by the internal arrangement of species from both taxa. *Psorophora* species were grouped together with *Culiseta* and *Coquillettidia*, and in a different clade from *Ochlerotatus* and *Aedes*. Yet *Aedeomyia* and *Uranotaenia* were more closely related to the *Ochlerotatus*+*Aedes* group than to the *Psorophora*, *Culiseta* and *Coquillettidia* group (Figure 3A).

### COI Analysis

Species from Argentina were placed within the correspondent genus along foreign mosquito species. Although these trees were broadly similar, some differences can be ascertained (Figure 3B and S2), being the tree drawn on the Maximum Likelihood method more fitted to classic taxonomy at genera level (Figure 3B). In this particular tree, genera members from the tribe Aedini were interspersed in several branches. *Culex* species were clustered and show some inconsistence of some particular sequences analyzed by Parsimony and Distance (Figure S2). All *Anopheles* species were grouped in a single set, thus reflecting a monophyletic origin in all the COI trees obtained. *Orthopodomyia* and *Toxorhynchites* were a sister group of *Uranotaenia*. Species of *Psorophora* were clustered together.

Sequences analysis permitted the interpretation of some relationships between native and foreign mosquitoes, which can be summarized as follows:

*Ochlerotatusalbifasciatus* and *O* *. crinifer* : These species are floodwater mosquitoes with a diapause in the egg stage. *O* *. albifasciatus* is a pest mosquito because females are aggressive biters and can act as vectors of *Western equine encephalitis virus* [27]; and it develops mainly in shallow puddles of grasslands and adults emerge in large numbers about 7 to 10 days in spring and 11 to 22 days in autumn after flooding of breeding places [28]. *O* *. crinifer* shares pools with the former species, but its larval density is usually lower and is more frequent in wooden areas [29], team personal observations]. The congeneric species more closely grouped with both mosquitoes, as seen in the 18S rDNA-based cladogram, were *O* *. sollicitans* , *O* *. triseriatus* and *O* *. canadensis* . COI-based topologies resulted in a close relationship between *O* *. albifasciatus* and *O* *. ventrovittis* , and *O* *. crinifer* and *O* *. atlanticus* . According to Crans’ life cycles classification [30], *O* *. atlanticus* is the species whose bionomics mostly resembles those of *O* *. albifasciatus* and *O* *. crinifer* , because they are multivoltine, floodwater mosquitoes. At least some populations of *O* *. albifasciatus* are resistant to salt-water [31], as *O* *. sollicitans* .

*Psorophora* *cyanescens* : This is also a floodwater species which larvae occurs in pools filled with spring and summer rainfall, associated with *O* *. albifasciatus* [32]. Females persistently seek hosts for blood-feeding between November and March in other areas of Buenos Aires province and their presence can coincide with *P* *. ferox* and *P* *. ciliata* [33]. In our 18S rDNA analysis, *P* *. cyanescens* was related to *P* *. ferox* and *P* *. ciliata* , therefore, Maximum Likelihood tree reflects the bionomics of these species. In the COI trees, *P* *. cyanescens* was located close to *P* *. longipalpus* , independently of the estimating method, while the other species of the genus ( *P* *. columbiae* , *P* *. signipennis* and *P* *. insularia* ) were placed in a separate cluster. This assemblage within *Psorophora* agrees with the classification at the subgeneric level, since *P* *. columbiae* *,* *P* *. insularia* and *P* *. signipennis* belong to *Psorophora* (*Grabhamia*), while *P* *. cyanescens* and *P* *. longipalpus* belong to *Psorophora* (*Janthinosoma*).

*Culex* spp.: Poor intraspecific resolution was observed regarding this mosquito genus as expected using these markers. Neither the COI- nor the 18S rDNA-based topologies lead to unambiguous positions of each species of *Culex* (Figure 3), so we cannot establish any relationship among them with certainty. Low bootstrapping values indicate that there is not enough information either in COI or 18S rDNA to resolve *Culex* species. Harbach [34] pointed out that *Culex* contains a high degree of polymorphism and exceptional forms. All species in our samples were classified within the subgenus *Culex* (Culex), which is diversified to the highest degree at the Neotropical region [35]. Most of the taxonomic research on *Culex* was only directed to differentiate species instead of a thorough classification that reflects natural relationships [36]. Demari-Silva et al. [8] established the relationships between *Culex* species from Brazil and suggested that *C* *. dolosus* may comprise a species complex. Intraspecific variation may add further confusion during identification. In Argentina, the distribution and hybridization of the *Culex* *pipiens* subgroup (sensu Harbach [36]) members such as *C* *. pipiens* and *C* *. quinquefasciatus* were studied in previous works. Humeres et al. [37] provided genetic evidence about the subspecific status of *C* *. pipiens* and *C* *. quinquefasciatus* , and contributed with information about genetic distance and gene flow between these taxa as well. However, other authors considered both taxa as separate species. *C* *. quinquefasciatus* is distributed from the provinces of Buenos Aires and Mendoza northwards, while *C* *. pipiens* was found from Buenos Aires southwards to Santa Cruz province [38,39]. Also, hybrid forms have been found in the central area of the country between 30° and 33° S [40,41]. Finally, Micieli et al. [42] were able to identify *C* *. pipiens* form *molestus*, *C* *. quinquefasciatus* and hybrids between them by morphological criteria and high-resolution molecular markers. The status of the members of the *C* *. pipiens* complex is still controversial, since some researchers consider *C* *. pipiens* and *C* *. quinquefasciatus* different species [36,42], while others ascribed them as subspecies of *C* *. pipiens* [43-46]. We consider all individuals in our samples as *C* *. pipiens* , because these were obtained within its geographical range [47], and did not attempt to identify subspecies or forms. *Culex* members probably could have been better discriminated using different gene polymorphisms, like the second intron of the acetylcholinesterase-2 (ace-2) locus [48], but this analysis was not the aim of this work. All the preceding analyses show that *Culex* species deserve further research using multidisciplinary procedures.

*Uranotaenia* *lowii* : Larvae of *U* *. lowii* are found in permanent, grassy shallow ponds, usually exposed to sunlight, from Southern USA to Argentina [49]. As females feed on amphibians, they are unimportant from a medical standpoint. This fact may explain in part the lack information on the phylogeny of the genera in the Neotropical region other than Lane [50], Galindo et al. [51] and Belkin et al. [52]. An interesting result of the 18S rDNA trees is the association between *Aedeomyia* and *Uranotaenia*, which suggests the relationship between them previously reported by Harbach and Kitching [25] (Figure S1).

In Mar del Plata we detected the presence of *C* *. pipiens* , *C* *. apicinus* , *C* *maxi* , *O* *. albifasciatus* , *O* *. scapularis* , *O* *. serratus* , *O* *. crinifer* , *P* *. ciliata* and *U* *. lowii* , which were reported as potential vectors of pathogens (http://www.cdc.gov/ncidod/dvbid/westnile/mosquitospecies) [53-57]. Several viruses transmitted by these mosquitoes were reported in Argentina, as well as the vectorial capacity of some of these species [58-64], justifying the importance of knowing the species present in this area of study and the increase health risk if we take into account the following issues. First, Mar del Plata increases its population during summer seasons, with a large movement of people around the country including areas of high prevalence of *Aedes* (=*Stegomyia*) *aegypti* (L). Second, there is a relative proximity of this city with Buenos Aires and La Plata (located at 400 and 370 Km away, respectively, to the North), both with a significant abundance of *A. aegypti* [65,66]. Third, the latter showed a progressive advance southwards [67]. Fourth, the gradual increase of the average temperature in the region has led to the introduction and development of insects such as lepidopterans common in warmer areas [68]. Thus, we might think that Mar del Plata could be a potential source for the introduction and spread of agents of medical interest.

New diseases are constantly being discovered and are becoming more widely distributed with the increase in traveling, to and from tropical and disease-endemic countries. According to the Pan American Health Organization [69], the prevention or reduction of transmission of most diseases being vectorized by mosquitoes depend entirely on the control of mosquito vectors or the interruption of human-vector contact, for this reason, the correct identification of the potential vectors involved and their geographic distribution is a critical step, as well as the active monitoring and surveillance of the natural mosquito populations.

## Conclusions

In this paper the identification of mosquito species present in General Pueyrredon district was made for the first time. The identification by morphologic characters allowed us to determine the presence of 14 species in the sample area. On the other hand, the analysis based on molecular methodologies yielded expected results. We agree with Shepard et al. [5] regarding the usefulness and limitations of 18S rDNA sequence to evaluate phylogenetic relationships among mosquitoes. Both sequences demonstrated to be successful to cluster mosquitoes al genera level.

Data provided in the present work contribute to the knowledge of mosquito distribution in the southeast area of Argentina and provide for the first time 18S rDNA and COI genes sequences that will be able to be used in subsequent analysis.

## Supporting Information

Figure S1 **A, Maximum Parsimony; B, Neighbor-Joining estimation using 18S rDNA sequences retrieved as explained in methods.** Node numbers indicate bootstraps support. 5000 replicates were used in Parsimony and Neighbor-Joining estimation. Arrows indicates local mosquito species.
(TIF)

Figure S2 **A, Maximum Parsimony; B, Neighbor-Joining estimation using COI coding-sequence retrieved as explained in methods.** Node numbers indicate bootstraps support. 5000 replicates were used in Parsimony and Neighbor-Joining estimation. Arrows indicates local mosquito species.
(TIF)

Table S1 **18S rDNA sequences used in the molecular analysis.**
(DOC)

Table S2 **COI sequences used in the molecular analysis.**
(DOC)
