# Supplementary figures and images for: Distribution of Mosquitoes in the South East of Argentina and First Report on the Analysis Based on 18S rDNA and COI Sequences
Source: PLoS One. 2013 Sep 30;8(9):e75516. doi: 10.1371/journal.pone.0075516 (PMC3787072; doi:10.1371/journal.pone.0075516)

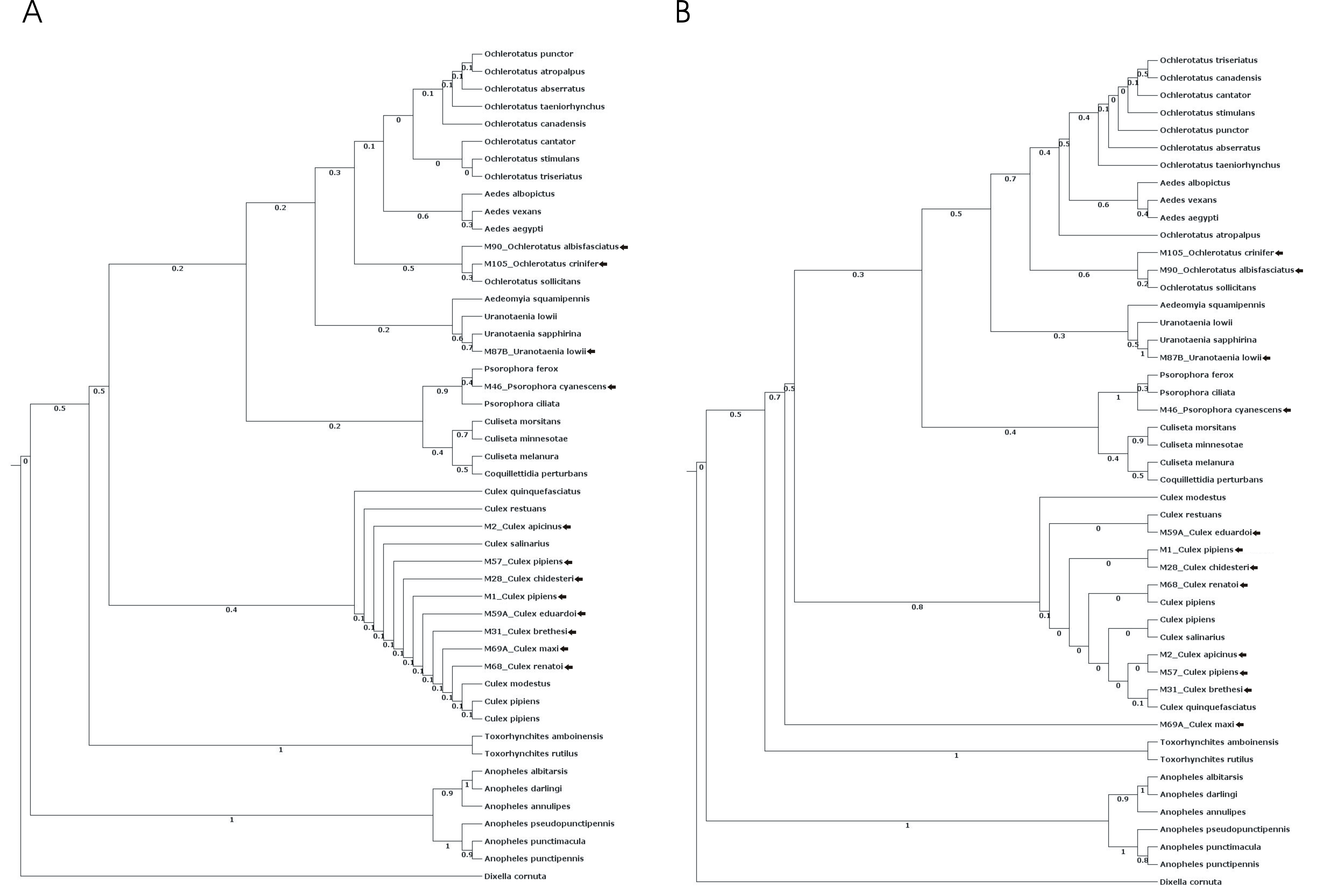

Supplement: Figure S1 — A, Maximum Parsimony; B, Neighbor-Joining estimation using 18S rDNA sequences retrieved as explained in methods. Node numbers indicate bootstraps support. 5000 replicates were used in Parsimony and Neighbor-Joining estimation. Arrows indicates local mosquito species. (TIF) [file pone.0075516.s001.tif]
