# Supplementary material for: Distribution of Mosquitoes in the South East of Argentina and First Report on the Analysis Based on 18S rDNA and COI Sequences
Source: PLoS One. 2013 Sep 30;8(9):e75516. doi: 10.1371/journal.pone.0075516 (PMC3787072; doi:10.1371/journal.pone.0075516)
Supplement: Table S1 — 18S rDNA sequences used in the molecular analysis. (DOC) [file pone.0075516.s003.doc]

Table S1: 18S rDNA sequences used in the molecular analysis

| **GenBank ID** | **Genus** | **Species** |
| --- | --- | --- |
| 5552 | *Aedes* | *albopictus* |
| 66269555 | *Aedes* | *vexans* |
| 66269556 | *Aedes* | *aegypti* |
| 66269542 | *Ochlerotatus* | *abserratus* |
| 365776008 | *Ochlerotatus* | *punctor* |
| 66269544 | *Ochlerotatus* | *cantator* |
| 66269545 | *Ochlerotatus* | *stimulans* |
| 66269548 | *Ochlerotatus* | *triseriatus* |
| 66269549 | *Ochlerotatus* | *canadensis* |
| 66269551 | *Ochlerotatus* | *sollicitans* |
| 66269552 | *Ochlerotatus* | *taeniorhynchus* |
| 66269554 | *Ochlerotatus* | *atropalpus* |
| 1230599 | *Toxorhynchites* | *amboinensis* |
| 66269571 | *Toxorhynchites* | *rutilus* |
| 66269561 | *Culex* | *pipiens* |
| 66269562 | *Culex* | *pipiens* |
| 66269563 | *Culex* | *quinquefasciatus* |
| 365776010 | *Culex* | *modestus* |
| 66269564 | *Culex* | *restuans* |
| 66269565 | *Culex* | *salinarius* |
| 66269558 | *Psorophora* | *ferox* |
| 66269559 | *Psorophora* | *ciliata* |
| 66269567 | *Culiseta* | *morsitans* |
| 66269568 | *Culiseta* | *minnesotae* |
| 66269569 | *Culiseta* | *melanura* |
| 66269570 | *Coquillettidia* | *perturbans* |
| 24430648 | *Anopheles* | *albitarsis* |
| 24430650 | *Anopheles* | *darlingi* |
| 5833121 | *Anopheles* | *annulipes* |
| 66269560 | *Uranotaenia* | *sapphirina* |
| 24430679 | *Uranotaenia* | *lowii* |
| 24430680 | *Aedeomyia* | *squamipennis* |
| 24430670 | *Anopheles* | *punctimacula* |
| 66269538 | *Anopheles* | *punctipennis* |
| 24430672 | *Anopheles* | *pseudopunctipennis* |
| 1230595 | *Dixella* | *cornuta* |
