# Supplementary material for: Distribution of Mosquitoes in the South East of Argentina and First Report on the Analysis Based on 18S rDNA and COI Sequences
Source: PLoS One. 2013 Sep 30;8(9):e75516. doi: 10.1371/journal.pone.0075516 (PMC3787072; doi:10.1371/journal.pone.0075516)
Supplement: Table S2 — COI sequences used in the molecular analysis. (DOC) [file pone.0075516.s004.doc]

Table S2: COI sequences used in the molecular analysis

| **GenBank ID** | **Genus** | **Species** |
| --- | --- | --- |
| 15724408 | *Aedes* | *aegypti* |
| 292397364 | *Aedes* | *vexans* |
| 364503658 | *Aedes* | *albopictus* |
| 397530423 | *Ochlerotatus* | *atlanticus* |
| 397530727 | *Ochlerotatus* | *sierrensis* |
| 397530729 | *Ochlerotatus* | *sierrensis* |
| 397530685 | *Ochlerotatus* | *monticola* |
| 397530449 | *Aedes* | *bahamensis* |
| 292397084 | *Ochlerotatus* | *aurifer* |
| 397530595 | *Ochlerotatus* | *fitchii* |
| 292397230 | *Ochlerotatus* | *provocans* |
| 292397102 | *Ochlerotatus* | *canadensis* |
| 292397266 | *Ochlerotatus* | *sollicitans* |
| 321135021 | *Ochlerotatus* | *sollicitans* |
| 292397086 | *Ochlerotatus* | *canadensis* |
| 397530453 | *Ochlerotatus* | *bicristatus* |
| 397530789 | *Ochlerotatus* | *ventrovittis* |
| 292397280 | *Ochlerotatus* | *stimulans* |
| 397530681 | *Ochlerotatus* | *melanimon* |
| 397739485 | *Ochlerotatus* | *mediovittatus* |
| 397530733 | *Ochlerotatus* | *squamiger* |
| 397530455 | *Ochlerotatus* | *burgeri* |
| 397530757 | *Ochlerotatus* | *triseriatus* |
| 397530615 | *Ochlerotatus* | *hendersoni* |
| 292397118 | *Ochlerotatus* | *cantator* |
| 397530485 | *Ochlerotatus* | *cataphylla* |
| 292397072 | *Ochlerotatus* | *atropalpus* |
| 397530443 | *Ochlerotatus* | *aurifer* |
| 397531421 | *Psorophora* | *columbiae* |
| 397531423 | *Psorophora* | *insularia* |
| 397531419 | *Psorophora* | *columbiae* |
| 397531425 | *Psorophora* | *longipalpus* |
| 397531431 | *Psorophora* | *signipennis* |
| 292397596 | *Culiseta* | *impatiens* |
| 292397598 | *Culiseta* | *inornata* |
| 292397612 | *Culiseta* | *morsitans* |
| 292397608 | *Culiseta* | *minnesotae* |
| 397531149 | *Culiseta* | *melanura* |
| 397531159 | *Culiseta* | *melanura* |
| 397739929 | *Culex* | *salinarius* |
| 397531045 | *Coquillettidia* | *perturbans* |
| 397531359 | *Mansonia* | *flaveola* |
| 218139346 | *Culex* | *theileri* |
| **GenBank ID** | **Genus** | **Species** |
| 397738404 | *Culex* | *theileri* |
| 218139350 | *Culex* | *theileri* |
| 292397554 | *Culex* | *restuans* |
| 397531079 | *Culex* | *bahamensis* |
| 373125604 | *Culex* | *pipiens* |
| 397531081 | *Culex* | *inhibitator* |
| 340050886 | *Culex* | *quinquefasciatus* |
| 397531097 | *Culex* | *taeniopus* |
| 397531075 | *Culex* | *apicalis* |
| 292397624 | *Orthopodomyia* | *alba* |
| 292397632 | *Toxorhynchites* | *rutilus* |
| 397531649 | *Uranotaenia* | *socialis* |
| 75756171 | *Anopheles* | *albitarsis* |
| 312064816 | *Anopheles* | *braziliensis* |
| 303303732 | *Anopheles* | *antunesi* |
| 309399337 | *Anopheles* | *deaneorum* |
| 292397444 | *Anopheles* | *quadrimaculatus* |
| 378788240 | *Anopheles* | *deaneorum* |
| 75756233 | *Anopheles* | *braziliensis* |
| 300490582 | *Anopheles* | *strodei* |
| 338856892 | *Anopheles* | *oswaldoi* |
| 402172979 | *Anopheles* | *punctimacula* |
| 338228369 | *Anopheles* | *lutzii* |
| 338856910 | *Anopheles* | *strodei* |
| 402172981 | *Anopheles* | *neomaculipalpus* |
| 338228371 | *Anopheles* | *lutzii* |
| 303303409 | *Anopheles* | *antunesi* |
| 312064796 | *Anopheles* | *triannulatus* |
| 75756229 | *Anopheles* | *darlingi* |
| 312064812 | *Anopheles* | *argyritarsis* |
| 312064836 | *Anopheles* | *pseudopunctipennis* |
| 338856812 | *Anopheles* | *argyritarsis* |
| 402172977 | *Anopheles* | *punctimacula* |
| 338228399 | *Anopheles* | *parvus* |
| 338856832 | *Anopheles* | *cruzii* |
| 401829744 | *Culicoides* | *obsoletus* |
